# *Gardnerella vaginalis* purulent meningitis in an adolescent male: a case report

**DOI:** 10.1186/s12883-022-02733-y

**Published:** 2022-06-11

**Authors:** Hongji Lu, Yaming Du, Tao Pan, Zheng Lou, Huiping Li, Yingdi Liao, Lixin Wang

**Affiliations:** 1 Department of Neurocritical Care, The Second Affiliated Hospital of Guangzhou University of Chinese Medicine, Guangzhou, 510120 China; 2 Department of Scientific Affairs, Hugobiotech Co., Ltd., Beijing, People’s Republic of China

**Keywords:** *Gardnerella vaginalis*, Meningitis, Cerebrospinal fluid, Metagenomic next-generation sequencing (mNGS), A case report

## Abstract

**Background:**

We report a rare case of *Gardnerella vaginalis* found in the cerebrospinal fluid of a young boy.

**Case presentation:**

A 14-year-old boy was admitted to hospital with headache, vomiting, fever, drowsiness and positive meningeal irritation signs on examination. Cerebrospinal fluid (CSF) shows white blood cell and protein were elevated, and glucose was low. Traditional aerobic and anaerobic culture of CSF did not grow any organisms. However, metagenomic next-generation sequencing (mNGS) reveals *G. vaginalis* in his CSF. The patient was diagnosed with purulent meningitis, and treated with intravenous meropenem and linezolid for a week, followed by oral administration of amoxicillin for two weeks. He recovered without sequelae.

**Conclusions:**

Purulent meningitis caused by *Gardnerella vaginalis* is extremely rare. Metagenomic next-generation sequencing of CSF should be highlighted for early diagnosis. With effective antibiotic treatment, the prognosis was excellent.

## Background

*G.vagianlis* is an anaerobic, bloodthirsty, difficult-to-culture, gram-negative or variable bacillus, originally discovered from female vagina [1]. A pathogen that should not be ignored in patients with bacteria vaginitis, it can lead to maternal infection and poor prognosis, such as chorioamnionitis, recurrent abortion and premature delivery [2–4]. Sexual contact is the main cause of infection in male. The majority of males infected are asymptomatic, but it is also associated with male urethritis and cystitis [5]. In addition to gynecological and urological diseases, *G. vaginalis* has been reported to cause other types of infection, such as bacteremia [6], multiple abscesses [7] and infective endocarditis [8]. However, to our knowledge, it is exceedingly rare that purulent meningitis was caused by *G. vaginalis*. Here, we reported the second case of meningitis in a boy caused by *G. vaginalis*.

## Case presentation

A 14-year-old boy was admitted to our neurological intensive care unit (NICU) with headache, vomiting, fever and drowsiness. He began to feel head pains six days before, and did not get better after antivirals and analgesics treatment at the clinic. Three days before, he began to vomit violently, mainly gastric contents, then he started to have fever, accompanied by chills, sweating, general fatigue, muscle and joint pain. The day before admission, he developed drowsiness. He had a girlfriend with a history vaginitis.

On admission, he was drowsy and insensitive to painful stimuli. His pupil diameters were 2.5 mm, and he was sensitive to direct and indirect light reflexes. Other cranial nerve examinations revealed no abnormality. The muscle strength of the limbs was level 5^−^, with normal muscle tone and sensation. Meningeal irritation signs were positive, and tendon reflexes of the extremities were normal. Computed tomography (CT) on head and chest were normal. Blood tests revealed that C-reactive protein was high at 211.95 mg/L (normal:0.00–6.00 mg/L), and procalcitonin was at 2.83 ng/ml (normal:0–0.05 ng/ml). Blood culture was sterile. Widal test, Weil-Felix test, detection of *Mycobacterium tuberculosis* DNA and indicators of immune function were normal. A lumbar puncture was performed, and the cerebrospinal fluid (CSF) was measured at 260mmH2O, slightly turbid. The CSF appeared a white blood cell count of 4620 × 10^6/L (90% neutrophils, 8% lymphocyte, 2% monocyte), protein of 2116 mg/L (normal:150-450 mg/L), and glucose of 2.42 mmol/L (normal:2.80–4.50 mmol/L).

**Fig. 1:**
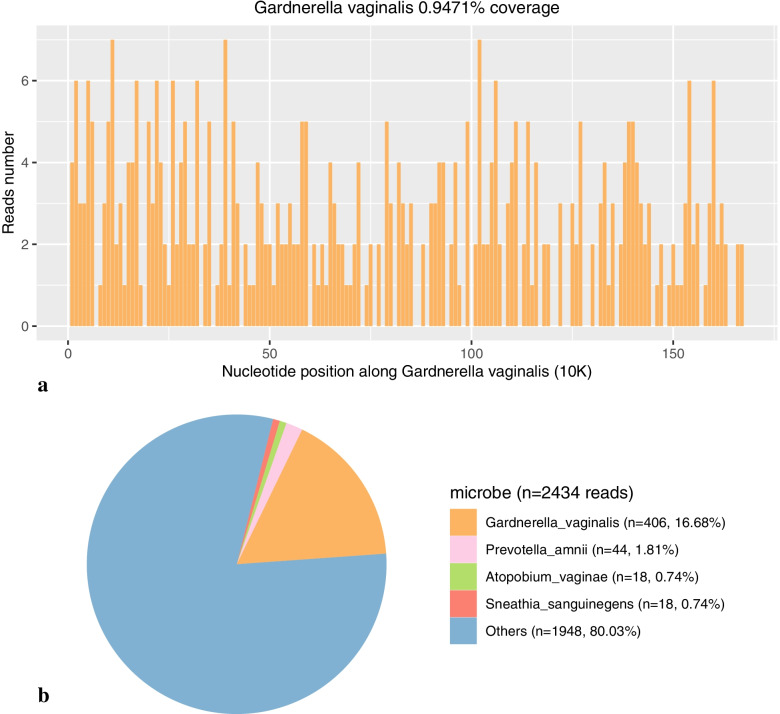
The coverage and abundance of *Gardnerella vaginalis* in the mNGS results. **a** the mNGS results had covered 0.9471% of the whole genome of *G. vaginalis,* with each bar representing the reads number aligned to a 10kbp region of the genome. **b** the 406 reads aligned to *G. vaginalis* genome occupied about 16% of the total 2434 reads for microbes

Aerobic and anaerobic cultures of the CSF via blood culture bottles (bioMerieux) at 35 ± 1.5 °C for 14 days did not demonstrate any organism(s) growth. However, mNGS (Hugo Biotech Co., Ltd.) revealed positive results from CSF, with *G. vaginalis* being the predominant microorganism, showing 406 unique reads of *G. vaginalis* with the coverage rate at 0.95% of its genome (Fig. 1). The results were confirmed by PCR tests (Hugo Biotech Co., Ltd.) with primers (forward, TTACTGGTGTATCACTGTAAGG; reverse, CCGTCACAGGCTGAACAGT) aligned to the 16S rRNA gene of *G. vaginalis.* Therefore, *G. vaginalis* was diagnosed as the causative pathogen.

According to the diagnostic result, we empirically administered intravenous meropenem (2 g, q8h) and linezolid (0.6 g, q12h) to the patient for a week. On the third day, with his headache alleviating and no vomiting, he was transferred out of the NICU. On the 6th day, the patient was awake, without headache, vomiting or fever. On the same day, magnetic resonance imaging (MRI) examination of his brain showed no abnormality. Reviewed examination of CSF showed that white blood cells at 10 × 10^6/L, protein level at 520 mg/L and glucose level at 2.91 mmol/L. Then, the patient was given oral amoxicillin for two weeks. After three months of follow-up, the patient had recovered completely.

## Discussion and conclusions

Although *G. vaginalis* is the main pathogen for female vaginitis, it can cause severe infections in other systems by damaging local tissues. Different from other anaerobic bacteria in vagina, *G. vaginalis* has higher pathogenicity due to its secretion of various virulence factors such as vaginolysin (VLY) and sialidase, as well as its biofilm formation. VLY secreted by *G. vaginalis* is a pore-forming toxin (PFT) and the high levels of VLY expression is related to high cytotoxicity [9, 10]. PFTs can destroy epithelial and endothelial cells in different tissues, causing the spread of bacteria to the corresponding tissues (eg., lung, brain) [11]. Studies had also shown that high level of sialidase produced by *G. vaginalis* can increase the risk of low birth weight and preterm birth [12]. Furthermore, it was demonstrated that *G. vaginalis* can adhere to vaginal epithelial cells and form thick biofilms. The formation of biofilm is an important factor in recurrent infections and antibiotic resistance [13]. In our case, the patient’s girlfriend had a history of vaginitis. we hypothesized that the patient contracted *G. vaginalis* from his sexual partner, and the highly expressed VLY could damage the brain endothelial cells, causing *G. vaginalis* to pass the blood-brain barrier. We had advised his girlfriend to have a physical examination.

Metronidazole is often used to treat bacterial vaginitis associated with *G. vaginalis*, and its cure rate is 80–90% [1]. Furthermore, previous studies had found that *G. vaginalis* was sensitive to erythromycin, chloramphenicol, ceftriaxone, ceftazidime ciprofloxacin and cefuroxime [14]. At present, β-lactams have been widely used to treat *G. vaginalis*-related bacteremia and other infections of the extra-vaginal systems [8, 10, 15]. In our case, the patient was treated with a combination of antibiotics (intravenous meropenem and linezolid), followed by oral amoxicillin. After sufficient antibiotics were given, the patient recovered without sequelae.

Three previous published cases of central nervous system (CNS) diseases caused by *G. vaginalis* are summarized in Table 1. Of the three patients, only one was diagnosed with meningitis. There was a five-day-old newborn, in whose CSF *G. vaginalis* was found, but with normal blood culture [15]. A young female patient presented with bacteremia and toxic encephalopathy. Brain MRI showed diffused white matter lesions. In her blood culture, *G. vaginalis*37, a subtype of *G.vaginalis*, was found to produce high concentrations of VLY which might disrupt the blood-brain barrier [10]. In addition, a male patient was infected by *G.vaginalis* from his girlfriend, resulting in multiple organ complications. His brain MRI revealed subacute septic embolism infracts of subcortical white matter at multiple sites [8].

**Table 1:** Three cases of central nervous system disease caused by *G.vaginalis*

Case No.	Sex	Diagnosis	Source of isolate	Treatment	Outcome
1 [15]	Female	Meningitis	CSF	Ampicillin, cefotaxime and netilmicin	Recovered
2 [10]	Female	Bacteremia, Toxic encephalopathy	Blood	Amoxicillin- clavulanate	Recovered
3 [8]	Male	Sepsis, Infective endocarditis and emboli in the kidney and brain	Blood	Metronidazole, ceftriaxone and erythromycin	Recovered

The small number of cases reported might be due to the lack of proper diagnostic tools. Recently, mNGS had been shown to successfully improve the diagnosis of neurological infections, with its higher sensitivity [16]. Our study suggested that the state-of-the-art mNGS technology showed high sensitivity in detecting *G. vaginalis* compared with other concurrent laboratory testing, and this target-independent identification of pathogens aided rapid pathogens identification and clinical treatment.

We reported a rare case of purulent meningitis due to *G. vaginalis*. After receiving the combined antibiotics treatment, the patient recovered with normal CSF and without discomfort. The case demonstrated the importance of timely diagnosis and treatments for meningitis caused by *G. vaginalis* infection. Accurate pathogenic microbiological examination, such as mNGS, that can provide qualitative and timely diagnosis for clinical, are beneficial to a favorable prognosis and thus recommended.

## Data Availability

All data related to this case report are documented within this manuscript.

## Abbreviations

CSF: Cerebrospinal fluid
mNGS: Metagenomic next-generation sequencing
NICU: Neurological intensive care unit
PFT: Pore-forming toxin
CT: Computed tomography
MRI: Magnetic resonance imaging
VLY: Vaginolysin
CNS: Central nervous system
